# Obtaining New Biocompatible Composite Materials with Antibacterial Properties Based on Diatomite and Biologically Active Compounds

**DOI:** 10.3390/molecules29071608

**Published:** 2024-04-03

**Authors:** Saule Kabieva, Gaziza Zhumanazarova, Rymgul Zhaslan, Gulistan Zhumabayeva, Arthur Ukhov, Dmitry Fedorishin, Alexander Gubankov, Farkhad Tarikhov, Ordabay Yerkhan, Kurzina Irina, Rakhmetulla Yerkassov, Abdigali Bakibaev

**Affiliations:** 1 Department of Chemical Technology and Ecology, Karaganda Industrial University, Temirtau 101400, Kazakhstan; kabieva.s@mail.ru (S.K.); rima93@list.ru (R.Z.); 2 Faculty of Natural Sciences, L.N. Gumilyov Eurasian National University, St. Satbaeva 2, Almaty District, Astana 010000, Kazakhstan; gulistan2009@mail.ru (G.Z.); kipchak_xan@mail.ru (O.Y.); erkass@mail.ru (R.Y.); 3 Chemical Faculty, National Research Tomsk State University, Arkady Ivanov St. 49, Tomsk 634028, Russia; artyryxov1@gmail.com (A.U.); strix187@yandex.ru (D.F.); 4.4.gub4nk0v@gmail.com (A.G.); farkhad.tarikhov@nu.edu.kz (F.T.); kurzina99@mail.ru (K.I.); bakibaev@mail.ru (A.B.)

**Keywords:** diatomite, betulin, chloramphenicol, tetrahydroxymethylglycoluril, biocompatible materials, antibacterial activity

## Abstract

This study aimed to create new composite materials based on diatomite—a non-organic porous compound—through its surface modification with bioactive organic compounds, both synthetic and natural. Chloramphenicol, tetrahydroxymethylglycoluril and betulin were used as modifying substances. Composite materials were obtained by covering the diatomite surface with bioactive substance compounds as a solution and material dispersion in it. The materials were characterized by IR spectroscopy, SEM and X-ray photoelectron spectroscopy. For the biocomposites, the hemolytic effect, plasma proteins’ adsorption on the surface and the antibacterial activity of the obtained materials were studied. Results show that the obtained materials are promising for medicine and agriculture.

## 1. Introduction

In the modern era of personalized medicine, implant material development has become an important issue which requires defining the specific balance of multiple parameters. These include composition, shape, structure, mechanical properties, biocompatibility, the ability to stimulate angiogenesis or bone formation, and antimicrobial activity. In implants or wound treatment materials, all these factors define the material ability to interact with surrounding tissue. Efficiently used bioactive materials can serve as a frame for new tissue growth [1].

On the other hand, while developing materials interacting with the milieu interieur, it is highly important to minimize their toxicity for body cells and tissues. However, many substances with required antibacterial properties are too toxic for the milieu interieur, and high antibacterial activity goes hand in hand with low biocompatibility. Due to this, the creation of materials combining both antibacterial properties and high biocompatibility is a difficult but important objective.

Hemocompatibility is one of the important aspects of biocompatibility. It includes thromboresistance—a biomaterial’s ability to prevent biomaterial thromb formation. This is a key feature of biocompatibility since foreign material interaction with blood may lead to coagulation or thromb formation. Hemocompatibility implies that the material does not negatively affect blood functions, composition changes and thromb formation [2].

The hemocompatibility of biomaterials depends on their physicochemical properties such as surface tension, free surface energy, roughness and hydrophilic capacity [2].

Another approach to studying hemocompatibility is related to the material surface electric charge. According to this model, blood protein adsorption occurs on abiotic surfaces, and the character of this adsorbed layer depends on the surface electric potential values and voltage difference. If the positive potential difference between the material and the blood is significant, thromb formation risk increases [3].

One of the materials that might possess the mentioned features is diatomite modified with bioactive compounds (**DA**). Diatomite is petrified residue of the diatomic plankton algae living in all bodies of water. Generally, diatomite almost completely consists of silicon dioxide (SiO_2_) [4]. **DA** is non-toxic and odorless, is present in nature in large quantities, is easily cleaned and is relatively cheap [5]. Diatomite has some unique features compared to other natural materials, including high porosity (10–100 nm), permeability, small particle size, large surface area (29 m^2^/g), high pore volume (0.09 cm^3^/g), low heat conductivity and chemical inertness [6]. Due to this, diatomite is used in various areas, for example, in construction, water filtration, agriculture, etc. The most well-known way of using it is as a contact insecticide in dry climates or as an ameliorant for soil and livestock or human food products [5].

However, the most promising prospect for diatomite is medicinal use as the base for potential systems of medicinal compound address delivery or as a component for bone implant creation. Diatomite has features stimulating bone tissue regeneration, such as large surface area, due to its porous structure and optimal roughness. These features are important factors for osteoblast adhesion and proliferation [4,7].

One of the necessary features of biomaterials is the ability to suppress the growth and development of bacteria entering a wound during surgery [8]. Bacteria entering a wound during surgical manipulations in the process of osteosynthesis may cause intrahospital and paraimplant infections. Such infections significantly decrease a patient’s quality of life and lead to more surgical operations with unpredictable outcomes.

However, **DA** itself does not possess antibacterial activity [4]. This is why in this study diatomite has been modified by coating its surface with organic compounds of natural and synthetic origin displaying antibacterial features. The high sorption capacity of diatomite allows it to provide long antibacterial compound emission from the biomaterial and, consequently, suppression of pathogenic wound microflora development. Due to this fact, diatomite was chosen as the base for biocomposites with antibacterial properties.

As mentioned above, the aim of this study was to research the antibacterial activity level of newly obtained biocompatible composite materials by modifying them with compositions of various nitrogen-containing compounds of natural and synthetic origin. In this study, the toxic effect of the obtained biocomposites was evaluated by studying their hemocompatibility, plasma protein adsorption and antibacterial activity. In this study, the impact of the biological effects of the composition of a number of synthetic and natural organic compounds applied onto diatomite in order to create promising biocomposite materials was researched. The frame base for the researched materials in the experiments was natural **DA** in two forms—intact (**IDA**) and cleaned (**CDA**), the latter obtained by boiling **DA** in hydrochloric acid [4].

## 2. Research Materials and Methods

Glycoluril was purchased from Novochem (Tomsk, Russia). All other chemicals were from Merck/Sigma–Aldrich (Darmstadt, Germany).

### 2.1. Diatomite (DA) Synthesis

This research used diatomite in two forms—intact diatomite (**IDA**) and diatomite that was additionally cleaned of involved cations and anions (**CDA**). The diatomite’s specific surface area was 29 m^2^/g, with SiO_2_ content approx. 90% by mass. Primary impurities were Al (2.53% by mass) and Fe (1.81% by mass), as well as Mg, Ca, K and Na which combined were approx. 2% by mass. This sort of diatomite is a promising material for obtaining various composites due to its surface and properties [9].

Diatomite cleaning was performed by boiling in 18% hydrochloric acid solution using the standard procedure [9]. To do this, a diatomite aliquot (m = 40 g) was submerged into hydrochloric acid solution (V = 300 mL). Then, the solution was brought to a boil and exposed for 24 h, and then the diatomite was filtered and dried at room temperature until reaching constant mass. The diatomites (**IDA** and **CDA**) were pressed into cylindrical pellets approx. 1 mm thick and calcinated at 600 °C.

### 2.2. Tetrahydroxymethylglycoluril (TGMGU) Synthesis

**Figure 1:**
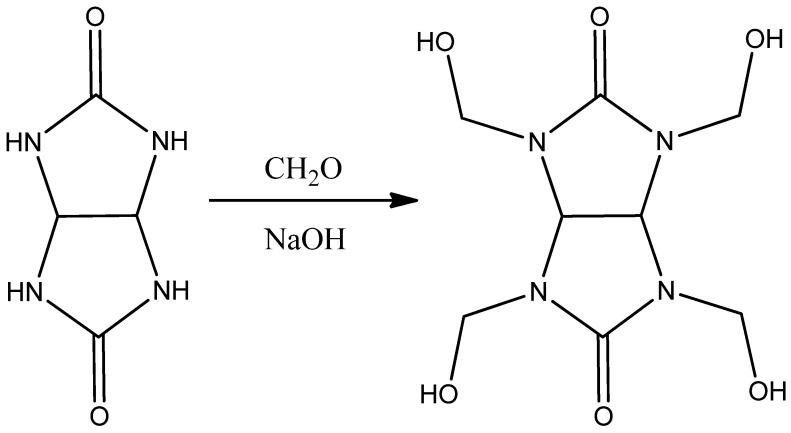
Method for obtaining **TGMGU**.

**Figure 2:**
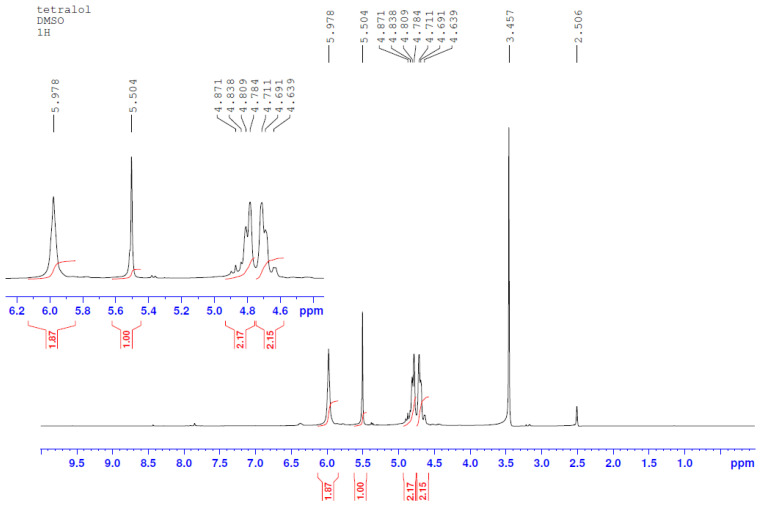
TGMGU 1H spectra.

Tetrahydroxymethylglycoluril (**TGMGU**) is a white powder highly soluble in water and dimethylformamide, but poorly soluble in most organic solvents. It was synthesized using the method given in [10] by condensing formaldehyde with glycoluril in an alkaline environment as shown in Figure 1:

The obtained substance was characterized using NMR spectroscopy:

Specter NMR ^1^H (Figure 2) (400 MHz, DMSO-d_6_), δ ppm Hz: 5.978 (c. 4H, OH), 5.504 (c. 2H, CH-CH), 4.80 (p. 2H, J = 11.2, N-CH_2_), 4.70 (p. 2H, J = 11.2, N-CH_2_).

**Figure 3:**
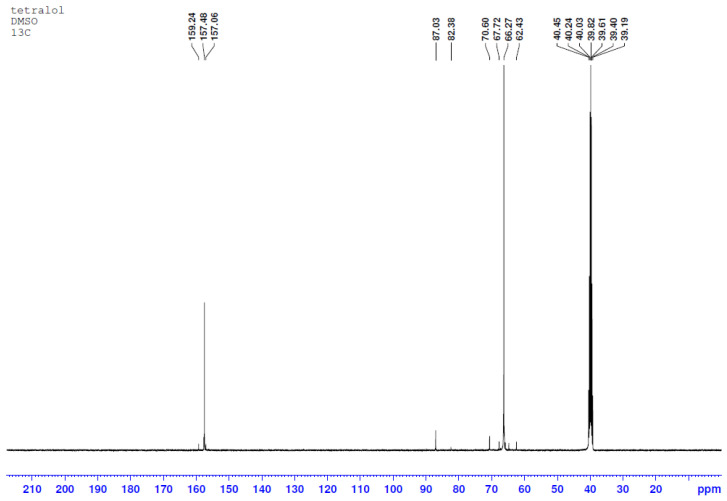
TGMGU 13C spectra.

Specter NMR ^13^C (Figure 3) (100 MHz, DMSO-d_6_), 8 ppm: 157.48 (C=O), 87.03 (CH-CH), 65.10 (N-CH_2_).

IR specter, v, cm^−1^: 1703 (C-O), 3273 (OH).

### 2.3. Betulin (BET) Separation

Betulin (**BET**) was separated through extraction from birch tree bark using the standard procedure [11]. The obtained substance was recrystallized from isopropyl alcohol and characterized using IR and NMR spectroscopy.

The IR specter (KBr, ν, cm^–1^) of betulin is characterized by absorption bands in the 3368 cm^–1^ area, which is typical for –OH groups, 1646 cm^–1^ (C=C) and others: 2968–2864 (C–H), 1450, 1372, 1105, 1029, 878.

^1^H NMR (400,17 MHz, CDCl_3_, δ, ppm): the weaker fields area has isopropenyl group proton signals (4.51 and 4.61 ppm); 3.26 and 3.73 (p, 2H, C_28_H_2_-OH); 3.10–3.14 (m, 1H, C_3_H-OH); 2.28–2.35 (m, 1H, C_19_–H); in the 0.79–2.00 ppm area there are lupane skeleton proton signals, where C-H group proton signals are found in betulin structure (6CH_3_, 10CH_2_, 5CH).

^13^C NMR (100,63 MHz, CDCl_3_, δ, m.f.): 150,51 (C-20); 109,71 (C-29); 78,98 (C-3); 64,46 (C-17); 60,51 (C-28); 55,28 (C-5); 50,38 (C-9); 48,74 (C-18); 47,79 (C-19); 42,71 (C-14); 40,91 (C-8); 38,87 (C-4); 38,69 (C-1); 37,30 (C-13); 37,15 (C-10); 34,22 (C-22); 33,98 (C-7); 29,74 (C-16); 29,16 (C-21); 27,99 (C-15); 27,38 (C-2); 27,04 (C-12); 25,37 (C-23); 25,19 (C-24); 20,83 (C-11); 19,09 (C-30); 18,30 (C-6); 16,12 (C-25); 15,98 (C-26); 15,38 (C-27).

### 2.4. Obtained Materials Characterization Methods

#### 2.4.1. Nuclear Magnetic Resonance (NMR) Spectroscopy

NMR specters of synthesized compounds were registered on an NMR spectrometer Bruker Avance 400 III HD in DMSO-d6 solution at 25 °C, working frequency at hydrogen nuclei—400 MHz, at 13C—100 MHz.

#### 2.4.2. Infrared (IR) Spectroscopy

Sample research was performed using IR spectroscopy with Fourier transform with an IR spectrometer Nicolet 6700, Thermo Fisher Scientific. Sample research was performed using the attenuated total reflectance method within a specter range between 400 and 4000 cm^–1^ with resolution 4 cm^–1^. Obtained reflectance specters were transformed into absorption bands using Kubelka–Munk transformation.

#### 2.4.3. Scanning Electron Microscopy (SEM) and Energy Dispersive X-ray Spectroscopy (EDS)

The JSM-IT200 is an easy-to-use compact scanning electron microscope, which enables the user to quickly and easily perform SEM analysis. The electron optics system, achieving a resolution of 3.0 nm at an accelerating voltage of 30 kV, is useful for a variety of applications, from high-resolution observation to EDS analysis. Specimen Exchange Navi, a beginner-friendly function, offers guided operation from sample loading to area search, and SEM image observation. Fully integrated EDS (silicon drift detector, resolution: 130 eV) includes “live EDS analysis”, displaying the chemical composition of the specimen while imaging. With the “Zeromag” function, sample navigation is easier than ever before. “Zeromag”, which links the SEM image with Holder Graphics or an optical CCD image, enables you to quickly locate areas for imaging and analysis.

#### 2.4.4. X-ray Photoelectron Spectroscopy (XPS) Analysis

For XPS analysis, we used a Nexsa X-Ray Photoelectron Spectrometer (XPS) by Thermo Scientific, which is a multi-technique surface chemistry analysis system. The main specifications of the Nexsa XPS system are as follows: sampling area: 3600 mm^2^ (samples must be smaller than a 60 × 60 mm sample holder); max. sample thickness: 20 mm; X-ray spot size: 10–400 µm (adjustable in 5 µm steps); analyzer type: 180°, double-focusing, hemispherical analyzer with 128-channel detector; depth profiling: EX06 monatomic ion source; and X-ray source type: monochromated low-power Al Ka X-ray source—1486.6 eV.

### 2.5. Biocomposite Hemocompatibility Evaluation

One of the ways to evaluate general cytotoxicity is to study hemolytical activity. Hemolysis is a process of hemoglobin release as a result of erythrolysis. The test for material hemolytical activity is based on erythrolysis and the hemoglobin dissociation degree during material contact with erythrocytes in vitro [12].

In order to evaluate hemocompatibility, whole hemostated blood from a healthy donor was used. After centrifuging, the blood was separated and erythromass was isolated. Obtained erythromass was diluted with sterile 1X PBS solution at 37 °C to a 1:9 ratio. Then, samples were placed into a standard 12-slot tray for cell cultivation and the obtained blood solution with PBS was poured at the ratio of 1 mln per 1 cm^2^ of sample surface area. As a positive control to define reaching 100% hemolysis, deionized water was used; as negative control, 1X PBS solution was used, which provides 0% hemolysis. During the experiment, **IDA** and **CDA** were used as control materials. Then, the tray with the samples was incubated in thermostat at 37 °C for 60 min. Next, the contents of the tray slots were transferred to centrifugal tubes and remaining erythrocytes were spun down by centrifugation for 5 min at 3000 rpm. Then, the supernatant was carefully removed and transferred to a standard 96-slot tray for further spectroscopy. The samples’ optical density was measured using the EIA reader TecanInfiniteF50 (Tecan Inc., Morrisville, NC, USA) at 492 nm.

The hemolysis percentage was the average value of three replications and was calculated using the formula given in GOST [12]:

$$\mathrm{Hemolysis},\;\%=\frac{OD_{\mathrm{test}}-OD_{\mathrm{control}}^{\mathrm{negative}}}{OD_{\mathrm{control}}^{\mathrm{positive}}-OD_{\mathrm{control}}^{\mathrm{negative}}}\times 100\%$$

### 2.6. Evaluation of Plasma Protein Adsorption by Biocomposites

In order to analyze plasma protein adsorption in studied samples, a modified method of solution depletion was used. This method includes two quantitative definitions of finding the protein concentration in blood plasma—before and after sample incubation [13].

We used the biuret reaction method to determine the amount of protein in solution. This method was chosen because of its simplicity and relative accuracy [14,15].

Initial plasma was separated from whole hemostated blood of a healthy donor by centrifugating. Then, the protein content in the plasma was found using the biuret reaction. Samples were filled with 2 mL of plasma and incubated at 37 °C in thermostat for 24 h. After incubation, the protein concentration was found a second time. The difference between the protein concentration of the initial plasma and that after incubation allows us to evaluate the protein adsorption rate—the greater this difference, the more protein that was adsorbed on the sample surface.

The interaction of blood serum proteins with cupric sulphate in a basic environment leads to the formation of complex compounds of purple color due to the proteins’ peptide bonds with copper ions. The intensity of the solution color depends on its protein concentration.

After the samples’ incubation and separation from the solution, the biuret reaction was carried out, followed by optical density measurement. To do this, 5.0 mL of working biuret reagent solution was added to 0.1 mL of blood plasma and carefully stirred, avoiding foam formation. The control experiment was performed simultaneously and 0.1 mL of 0.9% sodium chloride solution was used as control, then adding 5 mL of working biuret reagent solution. Then, 30 min later (but no later than 1 h), the solution optical density was measured using the EIA reader Tecan Infinite F50 (Tecan Inc., Morrisville, NC, USA) at 492 nm wavelength against control.

### 2.7. Biocomposite Antimicrobial Activity Evaluation

To evaluate the antibacterial activity of created samples, a standard disc diffusion method modified by the authors of this study was used. According to this method, the samples were placed on the surface of a high-density agar environment. **CHL**, **BET** and **TGMGU** (chloramphenicol, betulin and tetrahydroxymethylglycoluril) modifiers diffused into the environment, creating areas of bacteria growth suppression.

To study samples’ impact on the microflora, the *Escherichia coli* ATCC 25922 strain was used as test object. *Escherichia coli* was chosen as a model object because of the high importance of this microorganism for agriculture and medicine. The media used for *E. coli* cultivation were the standard LB media for these bacteria, containing peptone (10 g/L), yeast extract (5 g/L), sodium chloride (10 g/L) and microbiological agar (1.5–2% by volume). Incubation was carried out in thermostat at 37–38 °C for 24 h.

Each Petri dish with 15 mL of concentrated nutritious environment was inoculated with *E. coli* using lawn inoculation and pure bacteria culture. The volume of inoculum applied was 0.1 mL (0.5 McFarland’s standard, 10^8^ microorganisms/mL). Then, the sample was placed in the center of the dish. After incubation, the diameter of the zone with suppressed bacteria growth was measured to an accuracy of 0.1 mm. A large area of bacteria growth suppression implied a higher antibacterial activity of the sample. A bacteria growth inhibition area was considered one where growth was completely suppressed.

Samples without **CHL** in **IDA** and **CDA** were used as negative control. Samples modified by submersion into chloramphenicol solution (**IDA + CHL** and **CDA + CHL**) were used as positive control.

### 2.8. Statistical Analysis

Statistical analysis was performed using STATISTICA 8.0 for Windows (STATISTICA, RRID: SCR_014213). The Mann–Whitney test and t-test for independent groups were implemented. The data were checked for normality of distribution using the Shapiro–Wilk statistical criterion. Results were considered to be significant with *** *p* < 0.001, ** *p* < 0.01 and * *p* < 0.05.

## 3. Results and Discussion

Chloramphenicol (**CHL**) is an antibiotic used for treating bacterial infections. However, its use has been limited in recent decades due to its high systemic toxicity [16]. In this study, CHL was chosen as a model antibacterial compound because the test strain *E. coli* is highly sensitive to it.

Another substance used as a model antibacterial compound was tetrahydroxymethylglycoluril (**TGMGU**)—a nitrogen-containing heterocycle, one of bicyclic bis-ureas. As more bacteria strains become resistant to anti-bacterial compounds, those with biocidal properties become particularly interesting. **TGMGU** gradually releases formaldehyde, which causes its antibacterial properties. Also, **TGMGU** is used in medicine as a biomaterial component to improve fixation, keeping tissues’ and cells’ structure [13].

The third model compound was betulin (**BET**)—pentacyclic triterpenoid extracted from birch tree bark. Betulin and its derivatives possess various bioactive properties, including wound-healing, antibacterial, antitumoral, hypolipidemic, hepatoprotective and antiviral properties [17]. Betulin (**BET**) also can overcome resistance and induce apoptosis in malignized cells in various human cancer diseases. It displays antibacterial activity against *Bacillus subtilis* and *Escherichia coli*, and highly inhibits urease activity of *Helicobacter pylori* [17,18].

Moreover, **BET** wound-healing properties are caused by its ability to increase cell migration to damaged areas, increase their survival ability and stimulate collagen synthesis [19]. This wide range of biological activity suggests that **BET** can compensate for the toxic impact of antibacterial compounds, and combined with other antibacterial agents it can display synergy and increase their activity.

**Figure 4:**
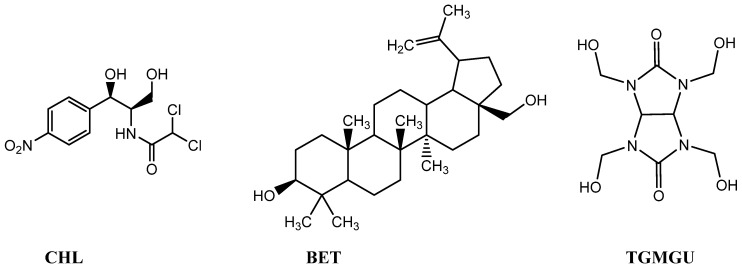
**DA** surface modifier structures: **CHL**—chloramphenicol; **BET**—betulin; **TGMGU**—tetrahydroxymethylglycoluril.

According to the stated objective, we studied the hemocompatibility and synergetic antibacterial activity of the composite materials. The following were used in this study as surface modifiers: chloramphenicol (**CHL**), betulin (**BET**) and tetrahydroxymethylglycoluril (**TGMGU**); their structures are shown in Figure 4.

**Figure 5:**
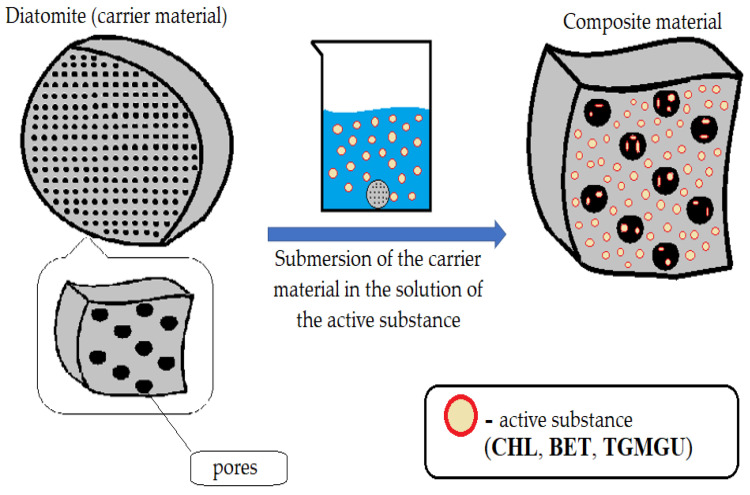
Illustration of bioactive substance (**CHL**, **BET**, **CHL + TGMGU**, **BET + TGMGU**) application onto **DA**.

In order to add modifiers, a **DA** sample was submerged into a working solution for 40 min (Figure 5). Working solutions were prepared as follows. For **CHL**, a 3% solution in ethyl alcohol was used to obtain the **IDA + CHL** and **CDA + CHL** composites. To obtain the **IDA + CHL + BET** and **CDA + CHL + BET** composites, 15 mg of **BET** was added to a 3% solution of **CHL** in ethanol. Solutions for obtaining composites with **TGMGU** were prepared in an ethanol solution with a concentration of each substance (**TGMGU** and **BET**) of 1 mg/1 mL. The resulting solutions were taken in an amount of 15 mL per sample. There also was a control experiment, where distilled water was used instead of bioactive solution. Obtained materials were completely dried at room temperature and studied for their biological activity.

For each obtained sample (**IDA** with **CHL**, **CHL + BET**, **TGMGU**, **TGMGU + BET** and **CDA** with **CHL**, **CHL + BET**, **TGMGU, TGMGU + BET**), the IR specter was measured. IR specters of substance (complex compounds) absorption were obtained using the IR Fourier spectrometer Nicolet 6700 with FTIR console on a diamond crystal (resolution 4 cm^−1^, 64 scans, range—4000–450 cm^−1^); no visible sample decomposition was observed. To obtain IR spectra, the samples were dried to a constant weight, and scanning was from the surface of the composites.

**Figure 6:**
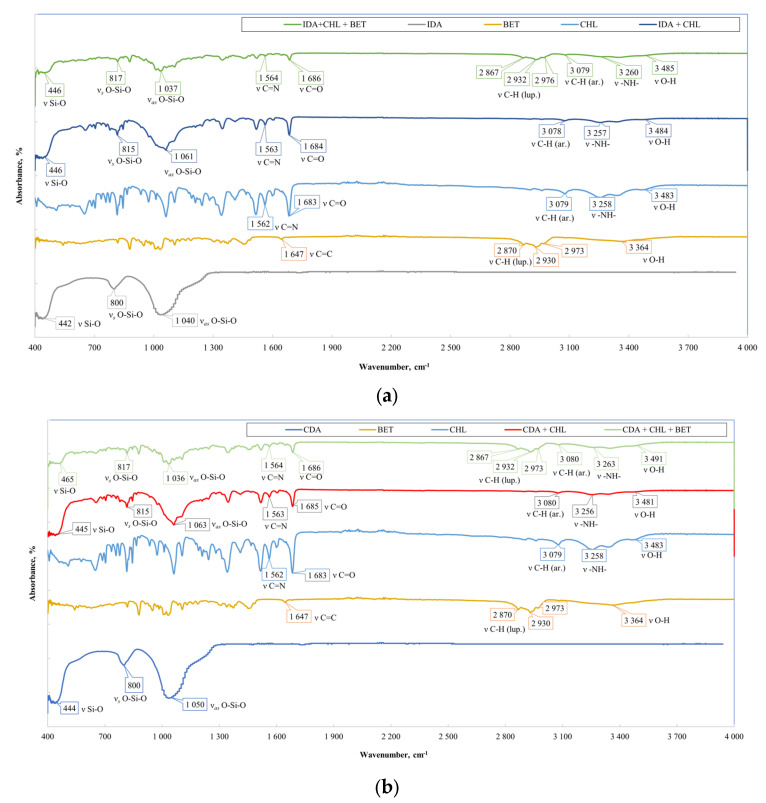
(**a**) Comparative specters of **CHL** samples and **CHL/BET** mixture on **IDA**. (**b**) Comparative specters of **CHL** samples and **CHL/BET** mixture on **CDA**.

The **IDA** with **CHL** sample specter (Figure 6a) showed the following absorption bands related to diatomite stretching vibrations—451, 816 and 1060 cm^−1^. The rest of the specter showed **CHL** absorption bands: stretching vibrations of OH-group were seen at 3480 cm^−1^, NH-groups of secondary amine at 3260 cm^−1^, C-H aromatic ring at 3080 cm^−1^ and C=O at 1680 cm^−1^, as well as nitrogroup stretching vibration absorption bands at 1560 cm^−1^. All the IR spectroscopy data set suggested **CHL** presence on **IDA** surface.

When analyzing the IR specter of **IDA** with **CHL** and **BET** fracture plane (Figure 6a), there were additional absorption bands typical for betulin—C-H stretching vibrations of the lupane skeleton at 2870, 2930 and 2970 cm^−1^. At 3480 cm^−1^, there was absorption band broadening as there was interference of the **BET**, **CHL** and **DA** OH-groups. All the IR spectroscopy data set proved **BET** presence in the composite. The IR spectra of the **CDA** samples (Figure 6b) showed a similar pattern.

**Figure 7:**
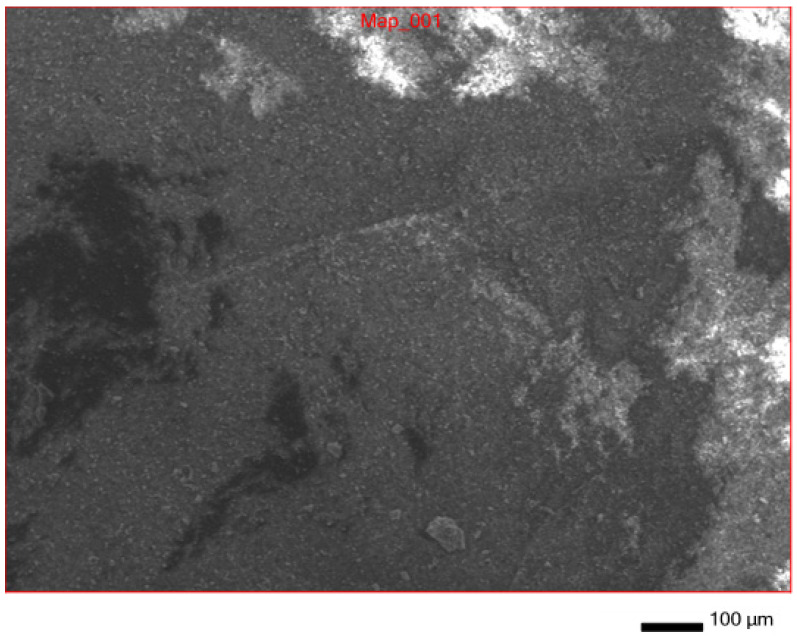
SEM surface of **IDA + CHL** composite.

**Figure 8:**
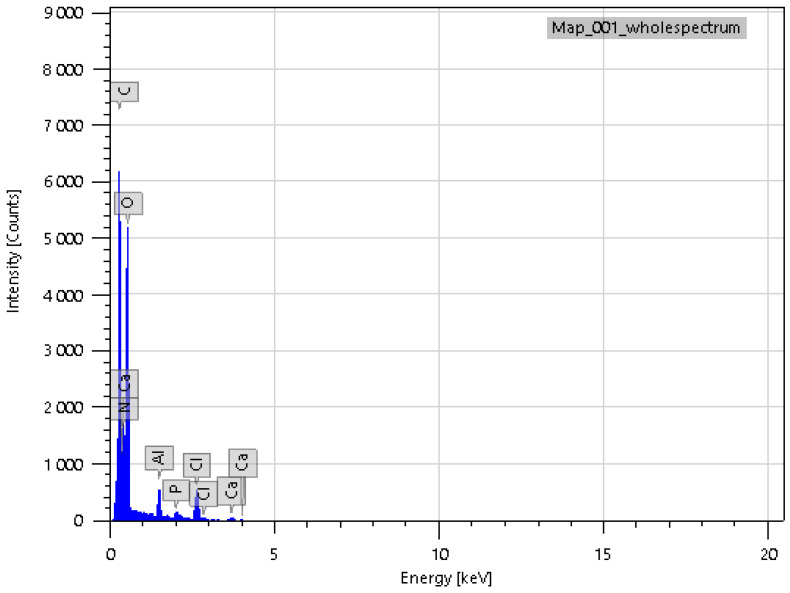
EDS of **IDA + CHL** sample surface.

In addition to IR spectroscopy, these samples were analyzed using SEM, EMF and XPS. To carry out the analysis, the samples were dried at room temperature to constant weight. The analysis was carried out from the surface of the scaffolds, which are circular in shape, 2 cm in diameter and 2 mm thick. The surface of the **IDA** is visible on the SEM images (Figure 7). An EDS was carried out from this surface (Figure 8), which showed that carbon is present on the surface in addition to the constituent elements of **DA** (silicon and oxygen).

**Figure 9:**
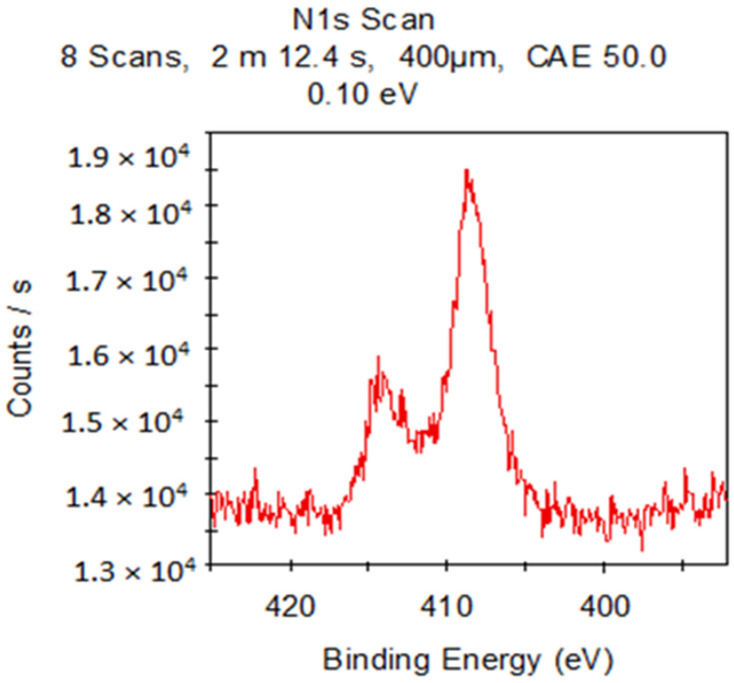
XPS of **IDA + CHL** sample.

The X-ray photoelectron spectroscopy (XPS) analysis of these composite materials provided information about the elemental composition and chemical bonding of the molecules. Since **IDA** and **CDA** contain only silicon and oxygen (other than metals), atoms will appear in the presence of organic substances (Figure 9), for example, nitrogen. The combination of these factors confirms the presence of **CHL** in the sample. Common acquisition parameters of the experiments were as follows: source gun type—Al K Alpha; spot size—400 µm; analyzer mode—CAE: Pass Energy 50.0 eV; and energy step size—0.100 eV.

**Figure 10:**
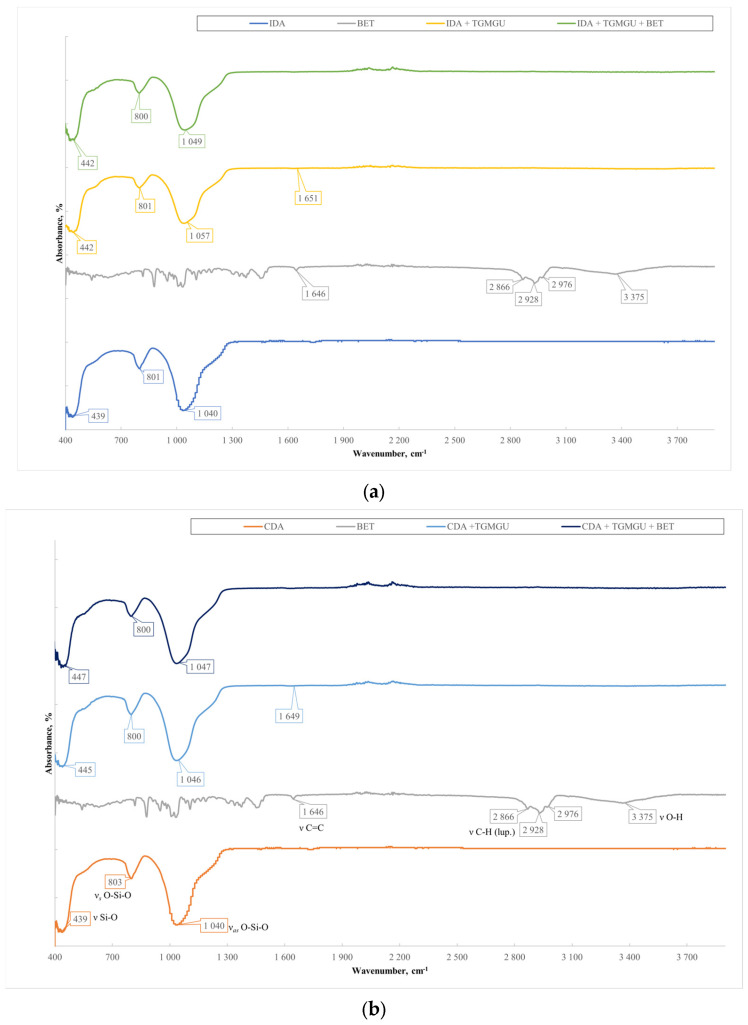
(**a**) Comparative specters of **TGMGU** and **TGMGU/BET** mixture materials on **IDA**. (**b**) Comparative specters of **TGMGU** and **TGMGU/BET** mixture materials on **CDA**.

Samples with cleaned diatomite provided a similar picture. All the typical absorption bands for the **CHL** and **BET** functional groups had the same positions (Figure 10).

Samples with **TGMGU** and **TGMGU/BET** mixtures were also analyzed using IR spectroscopy and X-ray photoelectron spectroscopy. Absorption maximums earlier identified for **DA** were also seen in the IR specters of these obtained materials. In the **IDA + TGMGU** sample section, there were no absorption bands typical for **TGMGU** (Figure 10a), but the samples still displayed a distinctive antibacterial activity (Table 6). The IR specter also had absorption bands at 1638 cm^−1^, which might be related to formaldehyde presence in the sample. The IR spectra of the **CDA** samples (Figure 6b) showed a similar pattern [20].

**Figure 11:**
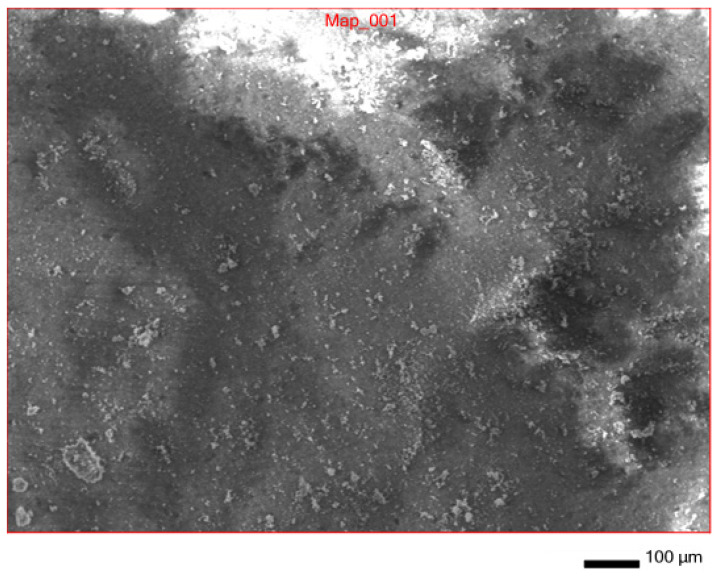
SEM surface of **IDA + TGMGU** composite.

**Figure 12:**
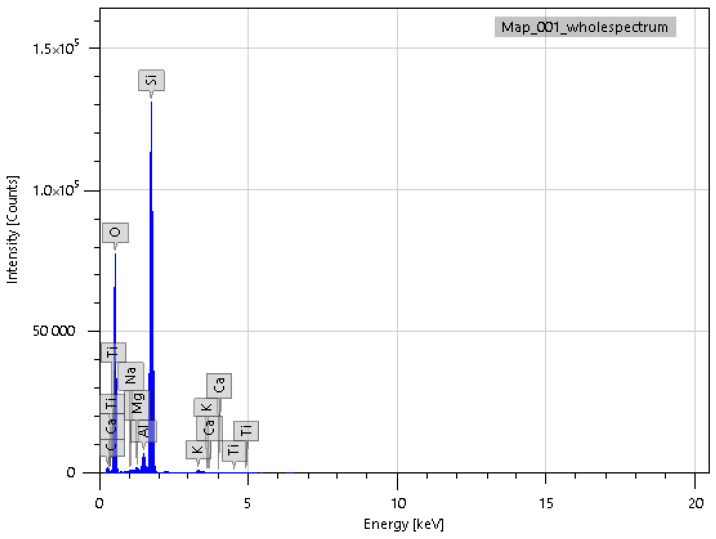
EDS of **IDA + TGMGU** sample surface.

The experiment observations were obviously related to the fact that **TGMGU** can extendedly separate formaldehyde in water solution [15]. Formed formaldehyde tends to bind with the **DA** surface and provide antibacterial effect, while the rest of the **TGMGU** does not remain due to high water solubility [21,22]. Another fact that suggests this is that in the SEM scan (Figure 11) no **TGMGU** or fragments thereof can be seen. The presence of free formaldehyde on the **IDA + TGMGU** sample surface can also be suggested from X-ray photoelectron spectroscopy scans (Figure 12). Considering the data (Figure 12) for carbon distribution on the **IDA** surface, a carbon-containing organic substance can be seen. Moreover, according to the SEM data, there were no nitrogen atoms on the **IDA + TGMGU** surface, as could be expected due to the use of **TGMGU**.

**Figure 13:**
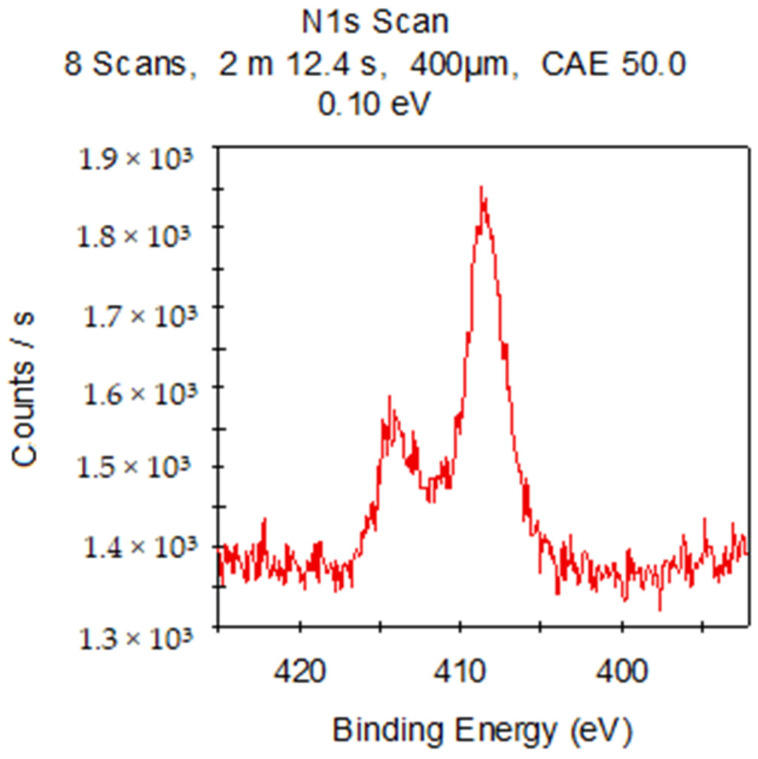
XPS of **IDA + TGMGU** sample.

In the X-ray photoelectron spectroscopy (XPS) analysis of these composite materials, in addition to the basic atoms (carbon and oxygen), the binding energy with nitrogen is observed (Figure 13).

Samples with cleaned diatomite produced a similar picture. The IR specters (Figure 10) did not show absorption bands; however, similar to the **IDA + TGMGU** composites, there were absorption bands typical for **DA**.

**Figure 14:**
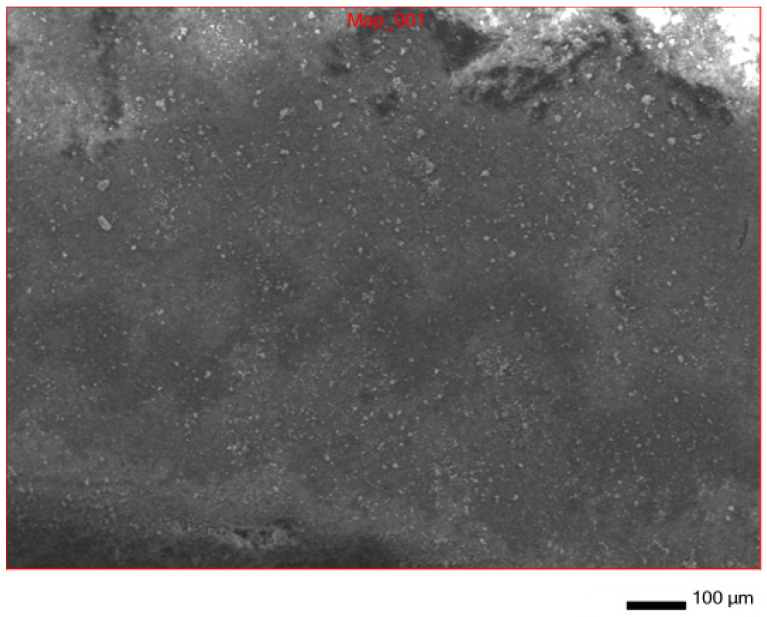
SEM scan of **CDA + TGMGU** composite surface.

**Figure 15:**
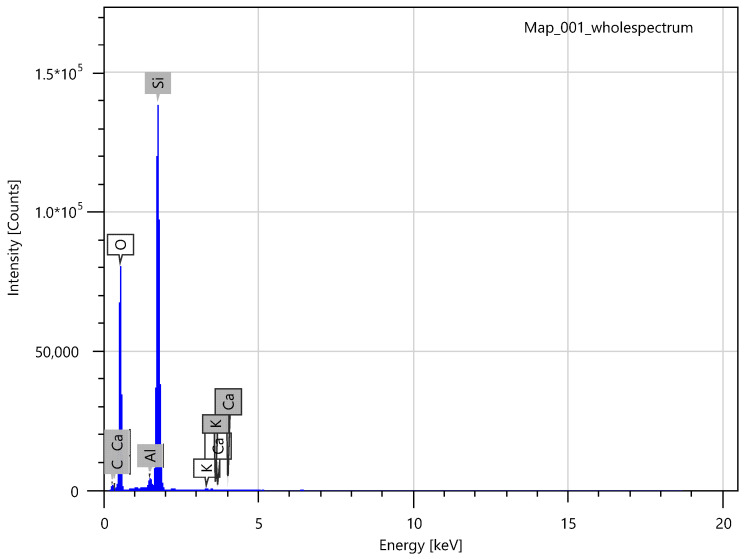
EDS scans of **CDA + TGMGU** sample surface.

These facts were also proven by SEM scan (Figure 14). Considering the X-ray photoelectron spectroscopy scans (Figure 15), carbon distribution on the surface can be seen, which suggests the presence of a carbon-containing organic substance (Figure 15), similar to the **IDA + TGMGU** composites.

**Figure 16:**
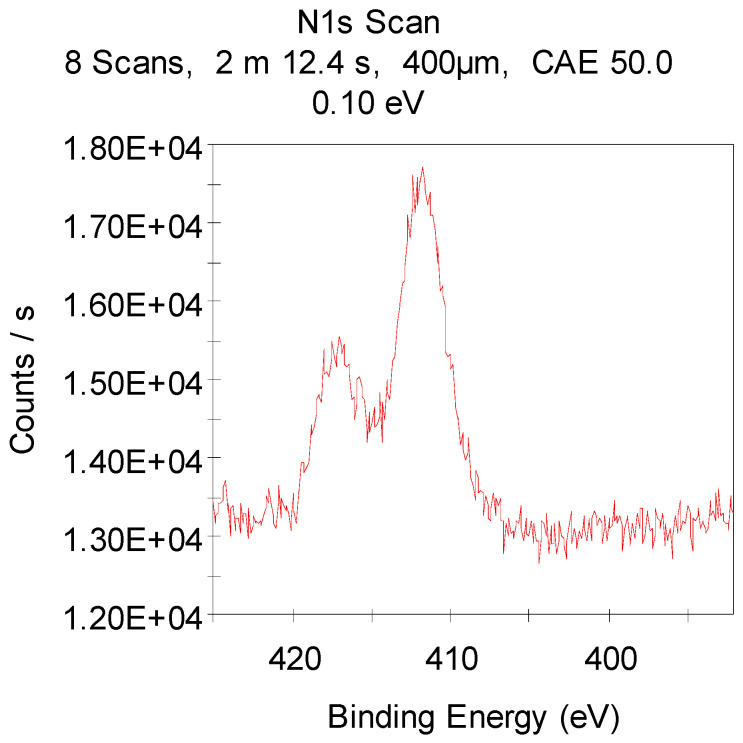
XPS of **CDA + TGMGU** sample.

The X-ray photoelectron spectroscopy (XPS) analysis of this composite produced a similar picture (Figure 16).

**Figure 17:**
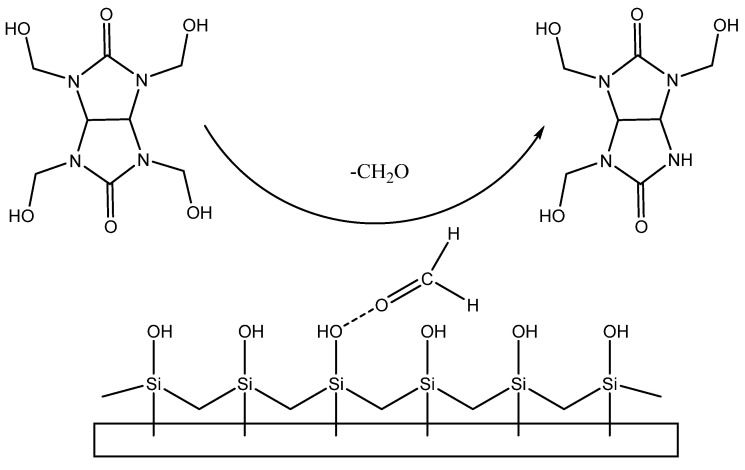
**TGMGU** and **DA** surface interaction pattern.

Based on the obtained data, a **TGMGU** and diatomite surface interaction pattern was produced (Figure 17).

**Figure 18:**
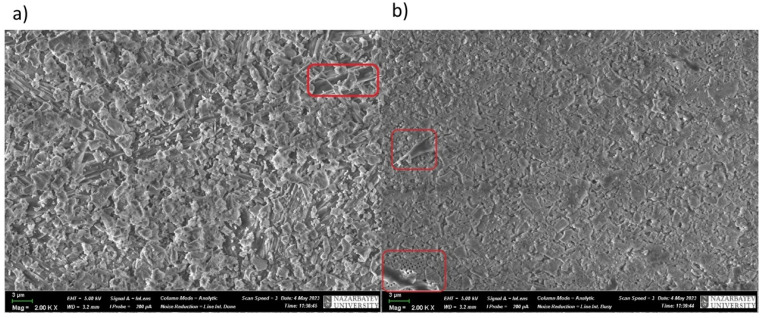
SEM scans of samples of **IDA + BET** (**a**) and **CDA + BET** (**b**).

The presence of **BET** on the composite surfaces can be suggested from SEM scans of **IDA + TGMGU + BET** (Figure 18a) and **CDA + TGMGU + BET** (Figure 18b). In these cases, **BET** is present as a dispersion in water solution and its crystals can be seen in the SEM scans—needle-shaped crystals on the surface marked with red rectangles.

After having studied the obtained composites **IDA + CHL**, **IDA + CHL + BET**, **IDA + TGMGU**, **IDA + TGMGU + BET**, **CDA + CHL**, **CDA + CHL + BET**, **CDA + TGMGU** and **CDA + TGMGU + BET,** we studied the toxic impact of the obtained biocomposites, which was estimated by studying their hemocompatibility, plasma protein adsorption and antibacterial activity.

One of the ways to estimate general material cytotoxicity is to study hemolytical activity.

Table 1 shows the hemolytical activity level estimation results for the sample groups **DA + CHL + BET**, which suggested that the modified **CHL** and **BET** samples did not display any hemolytical activity.

The absence of hemolytical activity in the studied composite samples may be related to the fact that during the **DA** treatment with **CHL** and **BET**, the base component’s porosity decreases. According to reference data, hemolysis on inert biomaterial surfaces is directly connected to plasma protein adsorption, and particularly to fibrinogen on the surface of the material being in contact with blood [23]. In other words, the more adsorption of plasma proteins there is, the greater the amount of hemolysis. Diatomite is a porous material with small pore size, at least they are smaller than the size of protein molecules, so plasma protein adsorption is insignificant on it [24]. Considering the abovementioned, it can be suggested that this is the reason for the low hemolysis level on these composites.

In order to determine whether the hemolytical activity level was connected to the plasma protein adsorption level in the studied samples, we studied the degree of the decrease in plasma proteins concentration after incubation with the samples obtained during the research using a modified solution depletion method.

As Table 2 suggests, all samples, including control ones (**IDA** and **CDA**), showed decreased protein in plasma after incubation, which was proven statistically (*p* < 0.05). Samples from the group **CDA + CHL**, as well as **IDA** and **CDA**, displayed the highest adsorption, but statistically no proven differences were found between them (*p* > 0.05). Other samples (**IDA + CHL**, **IDA + CHL + BET** and **CDA + CHL + BET**) also showed decreased protein content in solution (*p* < 0.05).

Supposedly, the low levels of the studied composites’ adsorption was related to the low hemolysis level and, consequently, low protein adsorption, the level of which was beyond the sensitivity of the method used. It was found that the betulin presence in the samples **IDA + CHL + BET** and **CDA + CHL + BET** also did not influence adsorption. Probably, the decrease in protein concentration after incubation may be connected not to surface adsorption but more to high porosity and the generally high sorption capacity of the studied samples.

As Table 3 suggests, the not modified with chloramphenicol (**CHL**) samples of the negative controls (**IDA** and **CDA**) did not display antibacterial effect in this method. Positive control group samples (**IDA + CHL** and **CDA + CHL)** displayed the highest antibacterial activity among all samples (51,0 and 52,1 mm, respectively). The antibacterial activity of the **CDA + CHL** samples was higher, but there were no statistically proven differences between them (*p* > 0.05). Betulin presence in samples did not influence the general antibacterial activity (*p* > 0.05).

According to our hypothesis, due to the high content of other natural mineral compounds and higher porosity, intact diatomite (**IDA**) would display higher antibacterial activity; however, no difference was found between intact and cleaned diatomite. Probably, this is connected to the high sensitivity to chloramphenicol (**CHL**) of the test strain used. The high background activity of chloramphenicol (**CHL**) did not allow us to find the fine differences in antibacterial activity in the created biocomposites due to the limitations of the disc diffusion method used.

Diatomites modified with **TGMGU** and **BET** displayed different levels of activity (Table 4).

As Table 4 suggests, the highest hemolysis level was shown by the **CDA + TGMGU + BET** samples (2.1122 ± 0.0026%) (*p* < 0.05). Other group samples did not display hemolytical activity (Table 4). Probably, the absence of hemolytical activity in these groups was connected to the high initial biocompatibility of diatomite, which is proven by reference data [4].

It was also found that a slightly higher hemolysis level was displayed by the **CDA** sample containing betulin (**CDA + TGMGU + BET**). Probably, this happened due to higher protein adsorption on the sample surface under betulin influence. However, it should be noted that the hemolysis level of biomaterials contacting the milieu interieur should not exceed 5% [12]. As the experiment shows, none of the studied samples exceeded this level, which shows that all the composite samples were hemocompatible.

It should be noted that the problem of unwanted blood clotting at contact with implanted materials and devices is still unsolved. The cause of this is the fact that healthy vessel endothelium has mechanisms resisting thromb formation, but foreign objects (i.e., foreign to the human body) do not have such defense mechanisms. Instead, biomaterials promote blood clotting through activating a number of interconnected processes including protein adsorption, thrombocyte and leukocyte adhesion, the production of thrombin and complement activation [23]. Due to this, the search for antibacterial substances that modify biocompatible materials but do not increase the hemolysis percentage is of high importance.

It should also be noted that the cytocompatibility control method tested in this study was successful as a reliable express method for general material cytotoxicity control. None of the samples exceeded the hemolysis level for medical materials contacting blood, according to ISO 10993-4:2017 [12].

As Table 5 suggests, the lowest adsorption level was displayed by the **CDA + TGMGU + BET** and **IDA + TGMGU + BET** samples, but no statistically proven differences between them were found (*p* > 0.05).

According to experiments previously performed in our study for hemocompatibility, the research sample **CDA + TGMGU + BET** displayed the highest hemolysis in the series of samples. However, the hemolysis level of this sample was 2.1122 ± 0.0026%, which suggests low hemolytical activity. However, the protein adsorption level for this sample was low. Probably, the treatment of the porous materials clogged their pores and reduced the sorption capacity, and the differences in adsorption level were connected directly to the samples’ physical structure.

Supposedly, a low adsorption level was related to a low hemolysis level and, consequently, low protein adsorption, the level of which was beyond the used method’s sensitivity. The presence of betulin (**BET**) in a sample also influences adsorption. Probably, the reduction in protein concentration after incubation was connected not to surface adsorption but more to high porosity and the generally high sorption capacity of the studied samples.

With respect to the antibacterial activity of composite **DA + TGMGU**, as Table 6 suggests, samples **IDA + TGMGU** and **CDA + TGMGU** also displayed antibacterial activity (27.9 and 31,9 mm, respectively), but no statistically proven differences between them were found (*p* > 0.05). But it can be noted that in the case of samples’ modification with betulin, the levels of activity changed. Samples **IDA + TGMGU + BET** and **CDA + TGMGU + BET** had growth suppression area diameters of 33,8 and 27,2 mm, respectively. The activity of sample **IDA + TGMGU + BET** was slightly higher, which was statistically proven (*p* < 0.05).

## 4. Conclusions

As we hypothesized, betulin as a bioactive compound will increase the antibacterial effect of **TGMGU**. This increase may also be due to the high porosity of intact diatomite (**IDA**) and the other natural mineral compounds present. In the case of intact diatomite (**IDA**) without betulin, the **TGMGU** activity is slightly lower, which was statistically proven (*p* < 0.05). However, the differences between the samples with and without betulin, although statistically proven, were not great in absolute values. Supposedly, this is connected to the test strain’s non-specific resistance to tetrahydroxymethylglycoluril. Probably, the test strain should be later replaced with another one, for example, *Bacillus subtilis,* which is subject to further research. It can also be assumed that the lack of additional antimicrobial effect in samples containing betulin may be due to the low solubility of betulin in water.

It should be noted that betulin is a natural compound with multiple potentially useful properties, although these are not currently researched. Supposedly, the increase in the biological activity of other active compounds is achieved not due to direct synergetic impact directly on bacteria, but due to the activation of complex interaction processes with other substances inside the human body and the enforcement of natural defense reactions. In the future, we plan to expand the number of test strains, antibacterial compounds and betulin derivatives used. These will be the subject of further research.

## Figures and Tables

**Table 1:** Hemocompatibility level for samples IDA, HA + CHL and HA + CHL + BET, CDA + CHL, CDA + CHL + BET, CDA + TGMGU, CDA + TGMGU + BET.

№	Sample	Hemolysis, %
**1**	IDA	0
**2**	CDA	0
**3**	IDA + CHL	0
**4**	CDA + CHL	0
**5**	IDA + CHL + BET	0
**6**	CDA + CHL + BET	0
**7**	CTRL 100%	100
**8**	CTRL 0%	0

Note: **IDA**—intact diatomite; **CDA**—cleaned diatomite; **CHL**—chloramphenicol; **CTRL**—control sample with pre-set hemolysis percentage.

**Table 2:** Level of plasma protein adsorption by samples IDA, HA + CHL and HA + CHL + BET, CDA + CHL, CDA + CHL + BET, CDA + TGMGU, CDA + TGMGU + BET.

№	Sample	Optical Density	Δ of Optical Density
**1**	IDA	0.1386 ± 0.0133	0.1227
**2**	CDA	0.1366 ± 0.0053	0.1247
**3**	IDA + CHL	0.1853 ± 0.0112	0.0847
**4**	CDA + CHL	0.1379 ± 0.0097	0.1321
**5**	IDA + CHL + BET	0.2049 ± 0.0318	0.0651
**6**	CDA + CHL + BET	0.1732 ± 0.0238	0.0968
**7**	CTRL (PBS)	0.1228 ± 0.0059	0.1472
**8**	CTRL (PLASMA)	0.2700 ± 0.0164	-

Note: **IDA**—intact diatomite; **CDA**—cleaned diatomite; **CHL**—chloramphenicol; **BET**—betulin; **CTRL (PBS)**—blank test; **CTRL (PLASMA)**—protein content in intact plasma.

**Table 3:** Antibacterial activity level of samples IDA, HA + CHL and HA + CHL + BET, CDA + CHL, CDA + CHL + BET, CDA + TGMGU, CDA + TGMGU + BET.

№	Sample	Growth Suppression Area Diameter, mm
**1**	IDA	0
**2**	CDA	0
**3**	IDA + CHL	51.0 ± 0.8
**4**	CDA + CHL	52.1 ± 0.5
**5**	IDA + CHL + BET	50.9 ± 0.6
**6**	CDA + CHL + BET	49.8 ± 1.1

Note: **IDA**—intact diatomite; **CDA**—cleaned diatomite; **CHL**—chloramphenicol; **BET**—betulin.

**Table 4:** Hemocompatibility level of samples IDA, HA + CHL and HA + CHL + BET, CDA + CHL, CDA + CHL + BET, CDA + TGMGU, CDA + TGMGU + BET.

№	Sample	Hemolysis, %
**1**	IDA	0
**2**	CDA	0
**3**	IDA + TGMGU	0
**4**	CDA+ TGMGU	0
**5**	IDA+ TGMGU +BET	0
**6**	CDA+ TGMGU +BET	2.1122 ± 0.0026
**7**	CTRL 100%	100
**8**	CTRL 0%	0

Note: **IDA**—intact diatomite; **CDA**—cleaned diatomite; **TGMGU**—tetrahydroxymethylglycoluril; **BET**—betulin.

**Table 5:** Protein content in plasma after incubation.

№	Sample	Optical Density	Δ Optical Density
**1**	IDA	0.1386 ± 0.0133	0.1227
**2**	CDA	0.1366 ± 0.0053	0.1247
**3**	IDA + TGMGU	0.1854 ± 0.0154	0.0846
**4**	CDA + TGMGU	0.1891 ± 0.0235	0.0809
**5**	IDA + TGMGU + BET	0.2423 ± 0.0147	0.0277
**6**	CDA + TGMGU + BET	0.2322 ± 0.0207	0.0378
**7**	CTRL (PBS)	0.1228 ± 0.0059	0.1472
**8**	CTRL (PLASMA)	0.2700 ± 0.0164	-

Note: **IDA**—intact diatomite; **CDA**—cleaned diatomite; **TGMGU**—tetrahydroxymethylglycoluril; **BET**—betulin; **CTRL (PBS)**—blank test; **CTRL (PLASMA)**—protein content in intact plasma.

**Table 6:** Antibacterial activity level of samples IDA, HA + CHL and HA + CHL + BET, CDA + CHL, CDA + CHL + BET, CDA + TGMGU, CDA + TGMGU + BET.

№	Sample	Growth Suppression Area Diameter, mm
**1**	IDA	No activity
**2**	CDA	No activity
**3**	IDA + TGMGU	27.9 ± 0.6
**4**	CDA + TGMGU	27.2 ± 1.0
**5**	IDA + TGMGU + BET	33.8 ± 1.2
**6**	CDA + TGMGU + BET	31.9 ± 1.1

Note: **IDA**—intact diatomite; **CDA**—cleaned diatomite; **TGMGU**—tetrahydroxymethylglycoluril; **BET**—betulin.

## Data Availability

Data are contained within the article.
